# Diagnosis and Management of Isolated Laryngeal Sarcoidosis: A Systematic Review

**DOI:** 10.1002/ohn.70129

**Published:** 2026-01-21

**Authors:** Raj Malhotra, Joseph Celidonio, Nicholas Hamilton, Alexandra Filipkowski, Kenneth Yan, Rachel Kaye

**Affiliations:** ^1^ Department of Otolaryngology–Head and Neck Surgery Rutgers New Jersey Medical School Newark New Jersey USA

**Keywords:** sarcoidosis, larynx

## Abstract

**Objective:**

This study reviews the existing literature on patients diagnosed with sarcoidosis isolated to the larynx to improve our understanding of the diagnosis and management of this rare condition.

**Data Sources:**

Embase, PubMed, Web of Science.

**Review Methods:**

We conducted a systematic review of published literature pertaining to patients diagnosed with isolated laryngeal sarcoidosis (LS). Demographics, symptomatology, diagnostic evaluation, management, and outcome details were recorded for patients diagnosed with isolated LS.

**Results:**

21 articles with 39 patients (11 male, 28 female, mean age: 33) were included (mean follow‐up: 33 months). The most common presentations were dyspnea (74%), dysphonia (67%), and dysphagia (49%). The supraglottic region was most involved (n = 37, 95%). Systemic steroids were the most used initial treatment, leading to symptom resolution in 27% of patients treated as such. 64% of all patients underwent further treatment for refractory symptoms, most commonly surgical excision with intralesional steroid injection. All patients (n = 8) who underwent initial treatment with systemic steroids followed by surgical excision with intralesional steroid injection experienced complete symptom resolution. Emergent tracheostomy was required in 8 (21%) patients during treatment and maintenance therapy was required in 11 (28%) patients. Long‐term outcomes (follow‐up mean: 33 months; range: 2‐192 months) showed 71% complete improvement, 19% partial symptom improvement, and 10% no improvement.

**Conclusion:**

Isolated LS most commonly affects middle‐aged women. Patients primed with a course of systemic steroids before undergoing surgical excision with intralesional steroid injection demonstrated the best outcomes. We recommend long‐term follow‐up of these patients for surveillance of symptoms and extra‐laryngeal sarcoidosis manifestations.

Sarcoidosis is a multisystem inflammatory disease characterized by non‐caseating granuloma formation in affected organs. Sarcoidosis most often affects the lungs with common extrapulmonary involvement seen in the skin, lymph nodes, and eyes. The earliest documented case of sarcoidosis was by Sir Jonathan Hutchinson in 1869.^1^, ^2^ 30 years later, Dr Boeck first described the histologic appearance of sarcoidosis, and thereafter, the first case of laryngeal involvement with histopathologic confirmation was reported in 1940.^3^, ^4^ Laryngeal involvement in systemic sarcoidosis is uncommon, reported in only 0.5% to 6% of cases.^5^, ^6^, ^7^ A diagnosis of isolated LS is made when biopsy‐proven sarcoidosis is limited to the larynx without the presence of imaging, biopsy, or clinical signs consistent with extra‐laryngeal sarcoidosis or an alternative diagnosis. Isolated LS is a rare, underreported, and understudied disease.

Patients with isolated LS commonly present with upper airway symptoms such as dyspnea and dysphonia, as well as dysphagia.^8^ These symptoms are due to granulomatous disease in the larynx causing edema of the upper airway, as well as secondary fibrosis of laryngeal structures. Diagnosis is made with laryngoscopic examination and biopsy of laryngeal lesions, as well as exclusion of other causes. The most common laryngeal subsite involved is the supraglottis, specifically the epiglottis. The edematous epiglottis has been described as sometimes taking on a characteristic turban‐like thickening.^9^

Different treatment modalities are utilized and include systemic immunosuppression and antineoplastics as well as targeted laryngeal interventions such as surgical excision and laryngeal steroid injections. However, currently there is no standard treatment algorithm for isolated LS. This is important to note as inadequate treatment can cause life‐threatening upper airway obstruction and respiratory compromise. This study aims to provide a systematic review of how different treatment modalities affect symptom resolution, the need for further treatment, and long‐term outcomes in patients with isolated LS, while also highlighting patterns in clinical presentation and diagnostic approaches.

## Methods

### Search Strategy

The guidelines from the Preferred Reporting Items for Systematic Reviews and Meta‐Analyses (PRISMA) were followed for this systematic review.^10^ The PubMed, Embase, and Web of Science databases were searched from inception to September 2024. The search terms used were as follows: laryngeal AND sarcoidosis.

### Study Selection

Two reviewers (RM and JC) independently screened all articles identified by the online database search. Rayyan, a systematic review collaboration platform, was used to facilitate review of records while blinded to the other screener.^11^ Duplicate articles were removed prior to review. Accessible articles that presented unique patient data regarding the diagnosis and management of isolated laryngeal sarcoidosis were included. All included patients required a histologic diagnosis of non‐caseating granulomas. If a study contained a mix of patients with isolated and non‐isolated LS, only those with isolated LS were included. Inclusion criteria were English language literature, studies retrievable with institutional journal access, and original patient data available (all ages included) on the diagnosis and management of isolated LS. Exclusion criteria included patients with a laryngeal manifestation of systemic sarcoidosis, any history of extra‐laryngeal sarcoidosis prior or after the time of LS diagnosis, or if the patient had biopsy‐proven sarcoidosis identified in another anatomical area at the time of the LS diagnosis. Patients with symptoms common in systemic sarcoidosis (fatigue, joint pain, weight loss) were evaluated independently for a laryngeal etiology to the symptom(s). In cases where the symptom(s) could not be attributed to LS and could represent occult systemic sarcoidosis, the patient was excluded. Any patient with a nonspecific imaging finding such as mediastinal lymphadenopathy, hilar lymphadenopathy, or pulmonary opacifications or consolidations was excluded due to possible undiagnosed systemic sarcoidosis. Discrepancies between the reviewers' evaluations were resolved through discussion with the senior author until a consensus was reached. Each article included was subjected to a bias assessment as described by the Agency for Healthcare Research and Quality (AHRQ).^12^ The AHRQ bias assessment evaluates articles for the following forms of bias: selection, performance, attrition, detection, and reporting.

### Data Extraction and Analysis

Data consolidation was achieved by compiling all included studies into a secure Microsoft Excel spreadsheet, which allowed organization of individual data points from each study. Data points were manually collected after review of each study, and compiled data for descriptive statistics were calculated in Microsoft Excel. The following data points were extracted from each study that met the inclusion criteria: patient demographics, presenting symptom(s), evaluation (laboratory, endoscopic, biopsy, imaging), areas of laryngeal involvement, treatment timeline and modalities, and follow‐up period. Laboratory evaluations included any serum or microbiology (bacterial, fungal, viral) workup. When reported, results from laryngoscopy were analyzed, including the laryngeal subsites involved and biopsy type and results. Any imaging, including chest X‐ray (CXR), CT sinus or neck, cervical MRI, or PET/CT was recorded. We assessed treatment effectiveness both individually and in relation to the sequence of treatments. Treatment modalities were categorized from initial to quaternary stages to evaluate the impact of each treatment step as well as the overall effectiveness of sequential therapy. A treatment modality was considered independent if the treatment team had sufficient time to assess the patient's response to the treatment and made the clinical assessment that additional treatment was necessary due to minimal, transient, or no treatment response. A partial treatment response was defined as subjective improvement without total resolution of symptoms after treatment. A complete treatment response was defined as total resolution of symptoms. Disease progression was measured by symptom progression and corroborated objectively via endoscopic visualization of the laryngeal lesions in some patients. Follow‐up time and the need for any maintenance interventions (repeat procedures or medications) were recorded. The flowchart figure was generated using Python executed in Google Colaboratory (Google Colab)—a cloud‐based computational environment (Google, 2025).^13^

## Results

### Article Extraction

**Figure 1:**
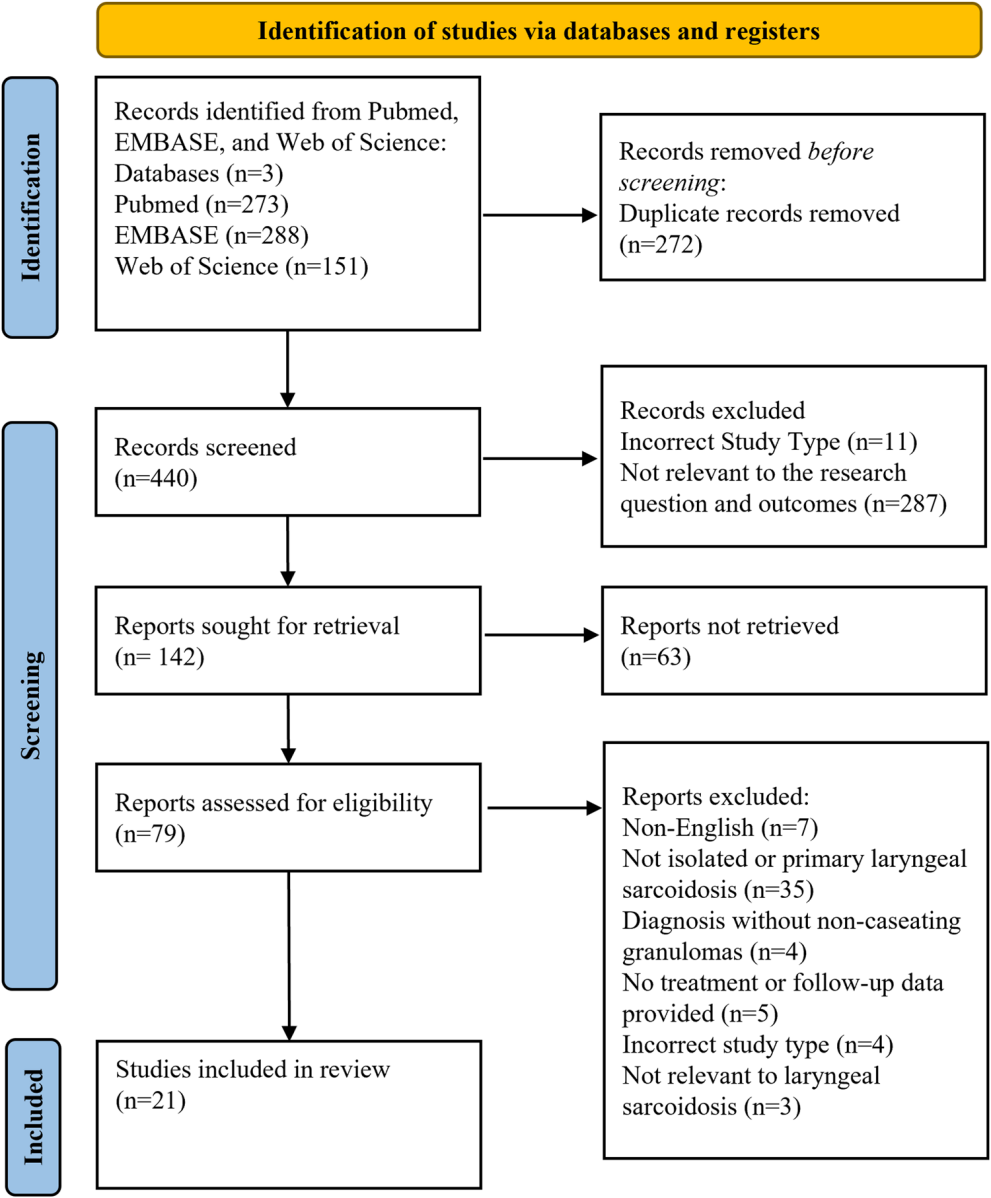
Article selection based on Preferred Reporting Items for Systematic Reviews and Meta‐Analyses (PRISMA) and search algorithm employed.

The initial database search identified 440 unique articles. Of the full texts screened, 21 articles were ultimately included (Figure 1).^7^, ^8^, ^14^, ^15^, ^16^, ^17^, ^18^, ^19^, ^20^, ^21^, ^22^, ^23^, ^24^, ^25^, ^26^, ^27^, ^28^, ^29^, ^30^, ^31^, ^32^ All 21 articles were included in the review according to the results of the bias assessment (Supplemental Table S1, available online).

### Patient Demographics

Among the 21 studies included in the analysis, 39 patients (11 male, 28 female) were identified as meeting the inclusion criteria (Supplemental Table S2, available online). The mean age of included patients was 33 (range: 12‐84). The most common presenting symptoms were dyspnea (n = 29, 74%), dysphonia (n = 26, 67%), dysphagia (n = 19, 49%), globus pharyngeus (n = 6, 15%), and cough (n = 6, 15%). Other less common symptoms included snoring, fatigue, sleep apnea, and weight loss (Table 1).

**Table 1:** Patient Demographics and Presenting Symptoms

	n (%)
Total	39
Age, mean [range]	33 [12–84]
Male	11 (28)
Female	28 (72)
Presenting symptoms	
Dyspnea	29 (74)
Dysphonia	26 (67)
Dysphagia	19 (49)
Globus pharyngeus	6 (15)
Cough	6 (15)
Snoring	4 (10)
Other^a^	

^a^ Other includes fatigue (n = 2), sleep apnea (n = 2), weight loss (n = 2), abdominal pain (n = 1), odynophagia (n = 1), and throat tightness (n = 1).

### Diagnostic Information

Diagnosis was often a multimodal approach involving laboratory evaluation, imaging, endoscopy, biopsy, and occasionally supplemental tests such as a pulmonary function test (PFT) or swallow study. The results of the various diagnostic tests used were recorded in 19 of the 39 (49%) patients (Table 2). The most common laboratory tests performed were angiotensin‐converting enzyme (ACE) levels, autoimmune workups, and inflammatory makers. ACE level was tested in 13 of 19 (68%) patients, of which 10 (77%) were within the reference range. An autoimmune workup including antinuclear antibody (ANA) titers, anti‐neutrophil cytoplasmic antibodies (ANCAs), anti‐DNA antibodies, SS‐A and SS‐B antibodies, or rheumatoid factor was carried out in 4 of 19 (21%) patients. Of these patients, none had positive findings. Inflammatory markers assessed include erythrocyte sedimentation rate (ESR) and C‐reactive protein (CRP). ESR was elevated in 2 out of 5 (40%) patients with reported ESR levels. CRP was not elevated in any patients with reported CRP levels. Complement level was normal in the one patient with reported levels.

**Table 2:** Laboratory and Microbiology Diagnostic Evaluation

	Normal/Negative n (%)^a^	Elevated/Positive n (%)^a^
Serum laboratory evaluation		
Angiotensin‐converting enzyme	10 (77)	3 (23)
Autoimmune workup^b^	4 (100)	0 (0)
Calcium	4 (100)	0 (0)
C‐reactive protein	3 (100)	0 (0)
Complement level	1 (100)	0 (0)
Erythrocyte sedimentation rate	3 (60)	2 (40)
IgG	1 (100)	0 (0)
IgM	1 (50)	1 (50)
IL‐2 Receptor	2 (100)	0 (0)
Liver function tests	3 (100)	0 (0)
Lysozyme	1 (100)	0 (0)
Thyroid function tests	1 (100)	0 (0)
White blood cell count	3 (100)	0 (0)
Microbiology		
Bacteria		
Acid‐fast bacilli smear	12 (100)	0 (0)
Bartonella henselae	1 (100)	0 (0)
Mycobacterium avium complex	1 (100)	0 (0)
Tuberculin skin test	2 (100)	0 (0)
TB (PPD, PCR, LJ‐medium)	5 (100)	0 (0)
Fungi		
Blastomycosis	2 (100)	0 (0)
Coccidiomycosis	2 (100)	0 (0)
Fungal culture	5 (100)	0 (0)
Histoplasmosis	2 (100)	0 (0)
Stains		
Methenamine silver stain	3 (100)	0 (0)

^a^ Row percentages are displayed.

^b^ Autoimmune workup included antinuclear antibody, anti‐neutrophil cytoplasmic antibody, anti‐DNA antibody, SS‐A antibody, and SS‐B antibody, rheumatoid factor.

Evaluation for microbiologic pathogens was conducted in 12 of 39 (31%) patients (Table 2). The most tested pathogen was Mycobacterium tuberculosis, for which all 12 patients who underwent microbiologic evaluation were assessed. The most common testing method was evaluation for acid‐fast bacilli (AFB) via smear or Ziehl‐Neelsen stain. Less common methods of testing for tuberculosis included tuberculin skin tests, polymerase chain reaction (PCR), and Löwenstein‐Jensen medium to detect Mycobacterium species. Several patients had multiple confirmatory tests to rule out tuberculosis. All 12 (100%) patients tested negative for tuberculosis. No fungal or viral pathogens were detected (Table 2).

In regard to diagnostic studies, 18 of 39 (46%) patients underwent at least one imaging study as part of their evaluation (Table 3). The most common imaging modalities performed were a chest radiograph (CXR) (n = 15), CT neck (n = 8), and MRI neck (n = 4). Patients with any hilar or mediastinal lymphadenopathy, or pulmonary infiltrates on CXR were excluded, hence all CXRs were normal. Of the 8 patients with documented CT neck results, 7 (88%) demonstrated laryngeal swelling and 1 (12%) was normal. Of the 4 patients who had a neck MRI, all demonstrated laryngeal swelling. PFTs were performed in 5 patients, 3 (60%) of whom were normal, and 2 (28%) demonstrated an upper airway obstructive pattern. A swallow study was conducted in 1 patient revealing delayed food transit with vallecular residue.

**Table 3:** Imaging Results

	n (%)
Chest X‐ray, total	15
Normal	15 (100)
CT Neck, total	8
Laryngeal swelling	7 (88)
Normal	1 (13)
MRI Neck, total	4
Laryngeal swelling	4 (100)
PET scan, total	3
Increased laryngeal uptake	2 (67)
Normal	1 (33)
CT Sinus, total	1
Normal	1 (100)

Regarding diagnostic modalities, all patients had an endoscopic biopsy performed. Biopsies were taken from diseased‐appearing subsites of the larynx. All patients had noncaseating granulomas. Laryngeal subsites involved were reported in all patients. The most common laryngeal subsite involved was the supraglottic region (n = 37, 95%). Of these patients, 17 (46%) involved epiglottis, 17 (46%) involved arytenoid cartilage, 16 (43%) involved aryepiglottic folds, and 5 (14%) involved false vocal cords. The glottis was involved in 4 (11%) patients, and the subglottic region was involved in 2 (5%) patients. The pyriform sinuses were involved in 3 (6%) patients.

### Treatments and Outcomes

**Figure 2:**
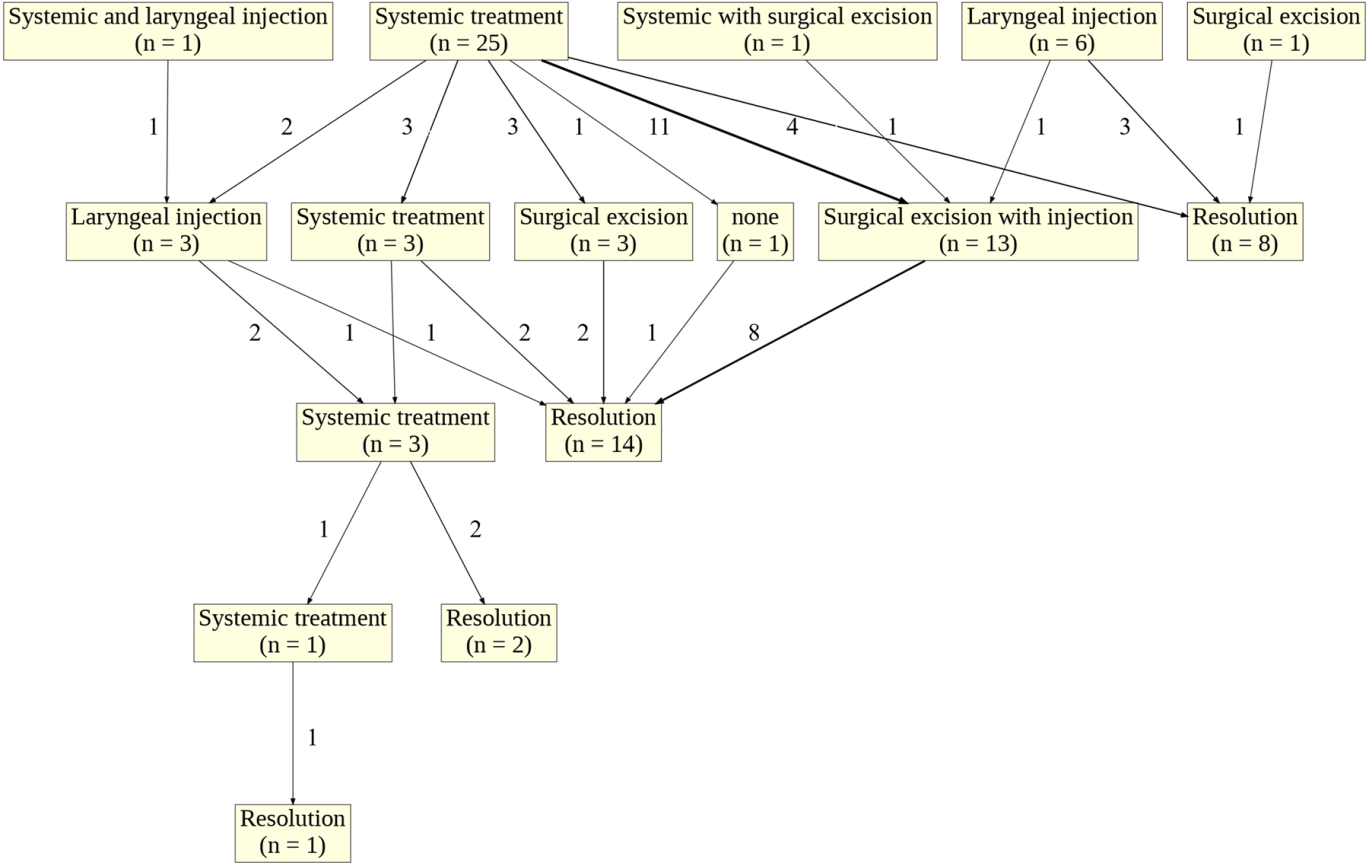
Flowchart depicting the number of patients who received each treatment modality. Up to four independent treatment modalities were recorded for each patient.

The most common primary treatment for isolated LS was systemic steroid administration. This treatment was given to 22 of the 39 (56%) patients with a documented initial treatment method (Figure 2). Among these patients, 27% of those who were treated with systemic steroids experienced marked improvement in their symptoms, while the remainder did not experience symptom improvement with steroid treatment. There were 9 (41%) patients who did not have outcomes recorded after the primary treatment with systemic steroids. Less commonly, targeted laryngeal interventions were utilized as initial treatment which included surgical excision, intralesional steroid injections, or mixed laryngeal and systemic interventions.

Secondary treatment was required in 25 (64%) patients (Figure 2). These included patients who showed no response to the initial treatment, or those with a partial or transient response. The most common secondary treatments were surgical excision with intralesional steroid injections (n = 13, 52%), systemic treatment (n = 3, 12%), laryngeal steroid injection (n = 3, 12%), and surgical excision (n = 3, 12%) (Table 4). These treatment methods showed complete improvement in 8 (62%), 2 (67%), 1 (33%), and 2 (67%) patients respectively. One patient with symptoms refractory to an intralesional steroid injection was successfully treated with CO_2_ laser division of a stenotic supraglottic area followed by topical application of mitomycin‐C to the surgical bed for 3 minutes.^21^ Another patient was treated off‐label with the antileprosy drug clofazimine after no response to systemic steroids, and experienced rapid and sustained symptom improvement over the four‐year follow‐up period.^27^

**Table 4:** Effectiveness of Treatment for Isolated Laryngeal Sarcoidosis

		Effective	
	Total, n^a^	Yes, n (%)	No, n (%)
Single intervention for initial management or refractory disease			
Laryngeal interventions			
Surgical excision	3	3 (100)	0 (0)
Surgical excision + intralesional steroid injection	9	8 (89)	1 (11)
Surgical excision + mitomycin‐C	1	1 (100)	0 (0)
Intralesional steroid injection	4	2 (50)	2 (50)
Intralesional steroid injection + dilation	1	0 (0)	1 (100)
Systemic intervention			
Systemic steroids	15	8 (53)	7 (47)
Immunosuppressant^b^	6	4 (67)	2 (33)
Clofazimine	1	1 (100)	0 (0)
Mixed laryngeal and systemic interventions			
Systemic steroids + intralesional steroid injection	1	0 (0)	1 (100)
Systemic steroids + surgical excision	1	0 (0)	1 (100)
Repeat interventions for refractory disease			
Laryngeal interventions			
Repeat intralesional steroid injection	4	2 (50)	2 (50)

^a^ Patients not accounted for in the row totals did not have outcomes documented after the respective treatment modality.

^b^ Immunosuppressants included methotrexate (n = 4), infliximab (n = 1), and sirolimus (n = 1).

Surgical resections, most commonly supraglottoplasty, were carried out using CO_2_ or YAG lasers for photoreduction, cold steel for debulking, and the “pepper‐pot” technique.^14^, ^15^, ^32^ Butler et al first described the “pepper‐pot” technique in 2010.^16^ This alternative to conventional debulking methods uses a “mucosa‐sparing multispot laser technique” to create laser ablation spots spaced 1 to 2 mm apart in mucosa to the depth of the sarcoid lesion. This technique preserves islands of mucosa and the function of affected areas while decreasing the volume of sarcoid lesions and minimizing scarring. This technique has since been employed in two large case series of 6 and 10 patients with lasting effects.^8^, ^16^

Tertiary treatment was required in 3 (12%) of the 25 patients who received secondary treatment (Figure 2). The most common tertiary treatment was methotrexate, which demonstrated a partial improvement in one patient and marked improvement in the other. The third patient was managed with off‐label sirolimus after no response to systemic steroids or methotrexate. Of these three patients, one underwent documented quaternary intervention. The patient was treated with an immunosuppressant (infliximab) and experienced complete improvement after poor response to systemic steroids, steroid injections, and methotrexate. Several patients (n = 11) required maintenance therapy, most commonly on systemic steroids (n = 6) (Table 5).

**Table 5:** Maintenance Therapies

	n (%)
Systemic steroids	6 (55)
Methotrexate	2 (18)
Intralesional steroid injections	1 (9)
Methotrexate + infliximab	1 (9)
Sirolimus	1 (9)

During the treatment course, 8 (21%) patients required an emergent tracheostomy. All tracheostomies were performed prior to treatment initiation. Of these patients, 7 had decannulation status documented. Successful decannulation was reported in 5 (71%) patients, 1 patient (14%) was not decannulated at 36‐month follow‐up, and 1 (14%) died from respiratory failure before treatment could be initiated. Among the patients successfully decannulated, three were initially treated with systemic steroids and two were initially treated with laryngeal interventions (surgical excision n = 1 and steroid injection n = 1). Secondary treatment was required in three of these patients. Two underwent laryngeal interventions (surgical excision n = 1, both injection and excision n = 1) and one underwent systemic treatment (methotrexate n = 1). One patient required tertiary treatment with sirolimus.

**Figure 3:**
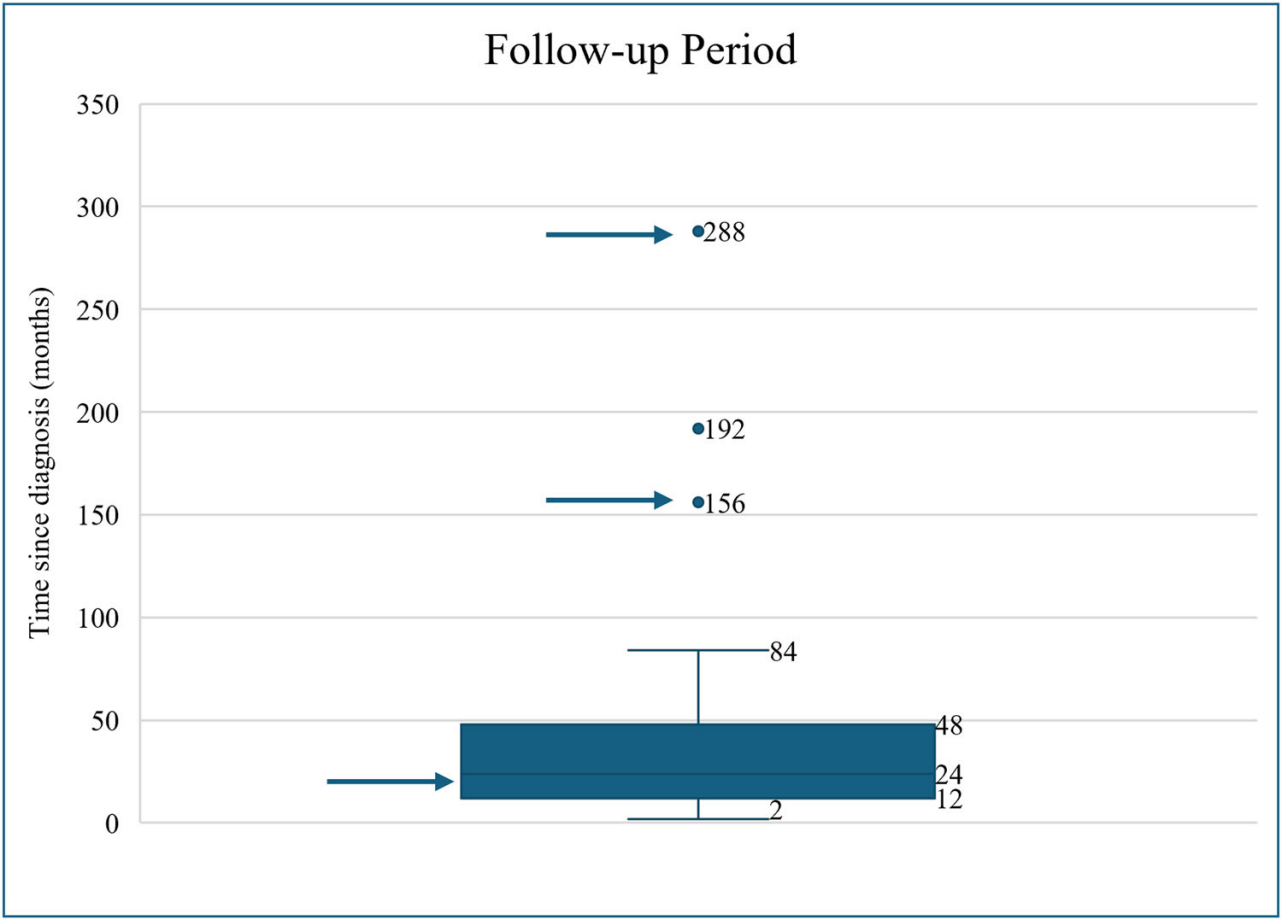
Box‐and‐whisker plot of the follow‐up period of the patient cohort. Arrows represent the follow‐up duration of the three patients diagnosed with extra‐laryngeal sarcoidosis during the follow‐up period.

The mean follow‐up time was 33 months (range: 2‐192). Long‐term outcomes demonstrated 71% complete improvement, 19% partial symptom improvement, and 10% without improvement. Notably, three patients were excluded from the analysis because they were diagnosed with extra‐laryngeal sarcoidosis during the follow‐up period. One of these patients was diagnosed with pulmonary sarcoidosis (288‐month follow‐up period), one was diagnosed with nasal, oral cavity, and pulmonary sarcoidosis (156‐month follow‐up period), and one was diagnosed with joint, nasal, and neurological sarcoidosis (24‐month follow‐up period). The length of two of these three patients’ follow‐up periods are outliers to the included patients (Figure 3).

## Discussion

Sarcoidosis is a multisystem granulomatous disorder classically characterized by bilateral hilar adenopathy, pulmonary opacities, and skin, joint, or eye lesions. The clinical manifestations of sarcoidosis can vary based on the specific organ system(s) involved. Head and neck involvement in sarcoidosis is present in 10% to 15% of cases with laryngeal involvement reported in only 0.5% to 5%.^33^, ^34^ Isolated laryngeal sarcoidosis is rare and likely underdiagnosed, making its prevalence unknown. Given that the literature on laryngeal sarcoidosis is limited to case reports and small series, this systematic review aims to provide greater insight into its diagnosis, management, and patient outcomes.

Consistent with previous reports on systemic sarcoidosis, our review found that middle‐aged women were the most affected demographic in isolated LS.^35^ Women were more than twice as likely to be diagnosed than men, and the mean age of all patients was 33. Patients with isolated LS most often presented with dyspnea, dysphonia, and/or dysphagia. The diagnosis of sarcoidosis is made using three primary features: clinical and radiographical evidence, histopathology demonstrating non‐caseating granulomas, and exclusion of other diagnoses. Diagnosis can be supported by imaging and laryngoscopy with biopsy. Consistent with previous reports, the supraglottic area is the most common laryngeal subsite involved.^8^ CT and MRI demonstrated laryngeal swelling in most patients. Alternative diagnoses that should be assessed include tuberculosis, fungal infection (especially in patients living in or previously in an endemic region), malignancy, and autoimmune conditions.

Our analysis demonstrated a limited diagnostic utility of laboratory evaluations in the diagnosis of isolated LS. Since Lieberman first reported elevated serum ACE levels in sarcoidosis patients in 1975, ACE elevations have been seen in up to 80% of untreated cases.^36^, ^37^ However, due to its poor sensitivity and specificity, serum ACE has a limited role in clinical practice. In our review, less than one quarter of patients with reported serum ACE level showed elevations, further underscoring its limited clinical utility in diagnosing or monitoring isolated LS.

Our analysis demonstrated significant overlap in treatment patterns of isolated LS and the more well‐established pulmonary sarcoidosis. Systemic steroids are considered first‐line treatment for symptomatic pulmonary sarcoidosis, with reports of steroid utility in treating sarcoidosis of the skin and heart as well.^38^, ^39^ Initial treatment with systemic steroids was effective in about one quarter of patients who initially received steroids. In patients with more severe pulmonary sarcoidosis, methotrexate is the preferred agent over steroids, which was also used in several patients in our cohort. Stratifying disease severity in pulmonary sarcoidosis is different than LS given the risk of acute airway compromise. LS severity was heterogeneously reported across the studies, but a severity stratification of LS would be helpful in guiding further research to optimize treatment, similar to prior work on pulmonary sarcoidosis.

Single or repeat intralesional steroid injections were also used as initial treatment and, although this treatment was utilized less commonly than systemic steroids, injections demonstrated similar outcomes. Thus, from our analysis, intralesional steroid injection was a reliable alternative to systemic steroids that can be considered for initial treatment, particularly in patients who cannot tolerate systemic steroids. We recommend caution in initially treating with surgical excision or non‐steroidal immunosuppressants, given the limited effectiveness in our cohort. Most patients needed secondary treatment for persistent or recurrent symptoms, most commonly surgical excision with intralesional steroid injections, which led to complete improvement in all patients without need for further treatment. It is important to note that most of these patients underwent previous treatment with systemic steroids. Given the high success rate of curative surgical excision combined with intralesional steroid injection following an initial course of systemic steroids, we recommend initiating treatment with systemic steroids to effectively prepare patients for a targeted, curative laryngeal intervention, if necessary.

Several novel medical and surgical therapies have been used successfully in the management of isolated laryngeal sarcoidosis. In 2000, Ridder et al successfully treated a patient who had symptoms refractory to systemic steroids with clofazimine, a riminophenazine antimycobacterial traditionally used to treat leprosy.^27^ At the time, clofazimine was thought to stimulate reticuloendothelial phagocytic cells and effectively managed several prior cases of orofacial granulomatous diseases.^40^, ^41^ In 2004, James et al used mitomycin, an antineoplastic antibiotic synthesized from *Streptomyces caespitosus* that has been shown to both inhibit fibroblast proliferation and prime fibroblasts to Fas receptor ligand‐mediated apoptosis.^42^, ^43^ Inhibition of the collagen meshwork created by fibroblasts prevents and destabilizes the formation of granulomas, which led to symptom improvement in this patient. Several other case reports discuss successful off‐label uses of antineoplastic agents such as the mTOR inhibitor sirolimus and Tumor Necrosis Factor‐Alpha (TNF‐α) inhibitor infliximab.^22^, ^25^ A new surgical technique described was the “pepper‐pot” technique to spare the laryngeal mucosa while debulking a sarcoidosis lesion.^16^ Further controlled studies beyond case reports are needed to understand the role that these medications and surgical techniques play in managing isolated LS compared to conventional steroid treatment.

Another important consideration in the treatment of isolated LS is the need for a tracheostomy, which about one quarter of our cohort required as an emergent procedure before starting treatment. Most patients were successfully decannulated following treatment, highlighting the clinical improvement achieved in most cases. A severity grading scale for patients with isolated LS may help identify patients at risk for airway compromise and who would benefit from early tracheostomy.

During long‐term follow‐up, most patients showed complete improvement. Due to limited follow‐up data and potential underreporting, the percentage of isolated LS cases that eventually progress to a systemic sarcoidosis diagnosis remains unknown. Three patients reviewed, but not included in our analysis, developed extra‐laryngeal sarcoidosis during follow‐up. Notably, two of these patients' follow‐up were among the longest in this review, highlighting the importance of long‐term surveillance in patients with isolated LS with high suspicion for extra‐laryngeal disease. We recommend ongoing surveillance with annual laryngoscopy and assessment for extra‐laryngeal manifestations of sarcoidosis. These may include an annual CXR, ocular examination, and maintaining a low threshold for biopsy of new‐onset arthralgia or skin lesions to evaluate for non‐caseating granulomas. Because the timeframe for potential development of systemic sarcoidosis following isolated LS remains unknown, and two patients developed systemic sarcoidosis after over a decade of follow‐up, we advise long‐term surveillance.

This review has several limitations. A major limitation is related to the rarity, likely underreporting, and limited long‐term follow‐up data associated with isolated LS. The rarity of LS also prevents accurate reporting of its prevalence, both in isolated and systemic cases. A centralized sarcoidosis database could mitigate these limitations; however, to the best of our knowledge this does not currently exist. There may also be reporting bias of cases that had a positive outcome, given many articles included were case reports or small case series. About half the patients included came from only three case series, which introduces a level of sampling bias. For a more thorough understanding of isolated LS management, a prospective, blinded trial to assess different treatment modalities is necessary. Due to inconsistent reporting among the included studies, we were unable to perform a sufficiently powered analysis of treatment regimen details including medication dosages and frequencies. These factors would be more appropriately evaluated in a controlled trial. Understandably, this is challenging given the rarity of the condition. Also, a larger sample size is necessary to analyze patient factors such as demographics and comorbidities that may influence the diagnosis, severity, and prognosis of isolated LS. Finally, findings from a repeat laryngoscopy were rarely documented, and so our assessment of treatment efficacy is based on symptom resolution rather than structural normality. Future research is necessary to address these limitations and improve the identification and treatment of patients with isolated LS.

## Conclusion

Isolated laryngeal sarcoidosis is a rare granulomatous disease, commonly affecting middle‐aged women and diagnosed based on clinical presentation, biopsy of laryngeal lesions, and exclusion of other etiologies. Although many patients require individualized treatment plans, we recommend initial treatment with systemic steroids. Most patients experience refractory symptoms, for which we recommend targeted laryngeal interventions such as surgical excision and laryngeal steroid injections, which resulted in long‐term improvement for the majority of patients. We recommend long‐term follow‐up of these patients, particularly for surveillance of symptoms and rare but important risk of extra‐laryngeal manifestations of sarcoidosis.

## Author Contributions

**Raj Malhotra**: design, conduct, analysis; **Joseph Celidonio**: conduct; **Nicholas Hamilton**: conduct; **Alexandra Filipkowski**: conduct; **Kenneth Yan**: design, analysis; **Rachel Kaye**: design, analysis.

## Disclosures

### Competing interests

None.

### Funding source

None.

## Supporting information


**Supplemental Table 1.** AHRQ (Agency for Healthcare Research and Quality) bias assessment of included articles.

**Supplemental Table 2.** Characteristics of included studies.
