# Supplementary material for: Diagnosis and Management of Isolated Laryngeal Sarcoidosis: A Systematic Review
Source: Otolaryngol Head Neck Surg. 2026 Jan 21;174(4):871–80. doi: 10.1002/ohn.70129 (PMC13035014; doi:10.1002/ohn.70129)
Supplement: Supplementary file 1 — Supplemental Table 1. AHRQ (Agency for Healthcare Research and Quality) bias assessment of included articles. [file OHN-174-871-s001.docx]

| Study | Selection Bias | Performance Bias | Attrition Bias | Detection Bias | Reporting Bias |
| --- | --- | --- | --- | --- | --- |
| Ahmadi et al. | N/A - case report | Yes | N/A | Yes | Yes |
| Barreiro et al. | N/A - case report | Yes | N/A | Yes | Yes |
| Butler et al. | N/A - case series | Yes | N/A | Yes | Yes |
| Craig | N/A - case report | Yes | Follow-up period not specified | Yes | Yes |
| Farlow et al. | N/A - case series | Yes | N/A | Yes | Yes |
| Fortune et al. | N/A - case report | Yes | N/A | Yes | Yes |
| Hilal et al. | N/A - case report | Yes | Follow-up period not specified | Yes | Yes |
| James et al. | N/A - case report | Yes | N/A | Yes | Yes |
| Kelleher et al. | N/A - case report | Yes | N/A | Yes | Yes |
| Kenny et al. | N/A - case report | Yes | N/A | Yes | Yes |
| Mayerhoff et al. | N/A - case series | Yes | N/A | Yes | Yes |
| Neel et al. | N/A - case series | Yes | N/A | Yes | Yes |
| Plaschke et al. | N/A - case series | Yes | N/A | Yes | Yes |
| Ridder et al. | N/A - case report | Yes | N/A | Yes | Yes |
| Rotman et al. | N/A - case series | Yes | N/A | Yes | Yes |
| Stensig et al. | N/A - case report | Yes | N/A | Yes | Yes |
| Strychowsky et al. | N/A - case report | Yes | N/A | Yes | Yes |
| Tsubouchi et al. | N/A - case report | Yes | N/A | Yes | Yes |
| van den Broek et al. | N/A - case report | Yes | N/A | Yes | Yes |
| Vaz et al. | N/A - case report | Yes | Follow-up period not specified | Yes | Yes |
| Weisman et al. | N/A - case report | Yes | N/A | Yes | Yes |
