# Supplementary material for: Diagnosis and Management of Isolated Laryngeal Sarcoidosis: A Systematic Review
Source: Otolaryngol Head Neck Surg. 2026 Jan 21;174(4):871–80. doi: 10.1002/ohn.70129 (PMC13035014; doi:10.1002/ohn.70129)
Supplement: Supplementary file 2 — Supplemental Table 2. Characteristics of included studies. [file OHN-174-871-s002.docx]

**Supplemental Table 2.** Characteristics of included studies.

| Author | Year | Study Type | n* | Age Mean | Male/ Female (n) | Areas of Laryngeal Involvement | Tracheostomy Yes/No (n) | Treatment Modalities | Follow-up Period (months) | Long-term Outcomes | Maintenance Therapy |
| --- | --- | --- | --- | --- | --- | --- | --- | --- | --- | --- | --- |
| Ahmadi et al. | 2020 | Case Report | 1 | 19 | 1/0 | S (E, AE, False VC), Trachea | 1/0 | Steroids, debulking | 3 | Significant | None |
| Barreiro et al. | 2012 | Case Report | 1 | 20 | 0/1 | S (AE) | 0/1 | Steroids | 6 | Significant | None |
| Butler et al. | 2010 | Case Series | 6 | 29 | 1/5 | 6x S (4x E, 6x A, 4x AE, 2x false VC) | 1/5 | Steroids, injection, debulking | Mean 29 | 4x Moderate, 2x Significant | Steroids |
| Craig | 1965 | Case Report | 1 | 26 | 0/1 | S (AE, false VC), pyriform sinus, glottis | 0/1 | Steroids | NR | None | None |
| Farlow et al. | 2021 | Case Series | 1 | 58 | 0/1 | 1x S (1x A) | 0/1 | Injections | NR | Significant | Injections |
| Fortune et al. | 1998 | Case Report | 1 | 28 | 0/1 | S (E, A, AE) | 0/1 | Steroids | 3 | Significant | None |
| Hilal et al. | 2022 | Case Report | 1 | 51 | 0/1 | S (E, AE), G | 1/0 | Steroids | NR | Significant | None |
| James et al. | 2004 | Case Report | 1 | 60 | 0/1 | S (E, A) | 0/1 | Injection, debulking, mitomycin-C | 30 | Significant | None |
| Kelleher et al. | 2020 | Case Report | 1 | 15 | 0/1 | S (E, A) | 1/0 | Steroids, Methotrexate, Sirolimus | 12 | Significant | Yes, Sirolimus |
| Kenny et al. | 2000 | Case Report | 1 | 14 | 1/0 | S (E) | 0/1 | Steroids, repeat debulking, injection | 6 | Significant | None |
| Mayerhoff et al. | 2010 | Case Series | 2 | 31 | 0/2 | 2x S (1x E, 2x A, 2x AE), 1x pyriform sinus | 0/2 | Steroids, repeat injections, Methotrexate, Infliximab | Mean 21 | 2x Significant | 1x No, 1x Yes, Methotrexate + Infliximab |
| Neel et al. | 1982 | Case Series | 6 | 44 | 2/4 | 5x S, 1x subglottis | 3/3 | Steroids, injections, debulking, | 54 | 3x Significant, 2x None, 1x NR | None |
| Plaschke et al. | 2011 | Case Series | 2 | 34 | 2/0 | 2x S (2x E, 2x A, 2x AE) | 0/2 | Steroids, debulking | Mean 12 | 1x Moderate, 1x Significant | None |
| Ridder et al. | 2000 | Case Report | 1 | 20 | 1/0 | S (E, AE, false VC) | 0/1 | Steroids, clofazimine | 48 | Significant | None |
| Rotman et al. | 2021 | Case Series | 7 | 25 | 2/5 | 7x S | 0/7 | Steroids, Hydroxychloroquine, Methotrexate, injection, repeat debulking | Mean 63 | NR | None |
| Stensig et al. | 2022 | Case Report | 1 | 14 | 0/1 | S (E, A) | 0/1 | Methotrexate, debulking, | 12 | Significant | Yes, Methotrexate |
| Strychowsky et al. | 2015 | Case Report | 1 | 13 | 0/1 | S (E, A, AE) | 0/1 | Steroids, injection, Methotrexate | 6 | Significant | Methotrexate |
| Tsubouchi et al. | 2015 | Case Report | 1 | 49 | 0/1 | S (E, A, AE) | 0/1 | Steroids | 12 | Significant | None |
| van den Broek et al. | 2013 | Case Report | 1 | 35 | 0/1 | S, G, subglottis | 0/1 | Steroids | 2 | Significant | None |
| Vaz et al. | 2000 | Case Report | 1 | 84 | 0/1 | S (A), G | 0/1 | Died before treatment could be initiated | Deceased | Died from respiratory failure | N/A |
| Weisman et al. | 1980 | Case Series | 1 | 31 | 1/0 | S (E) | 0/1 | Steroids | 18 | Significant | None |

*Total patients (n) include only patients in each study with isolated laryngeal sarcoidosis.
